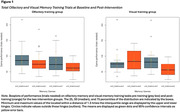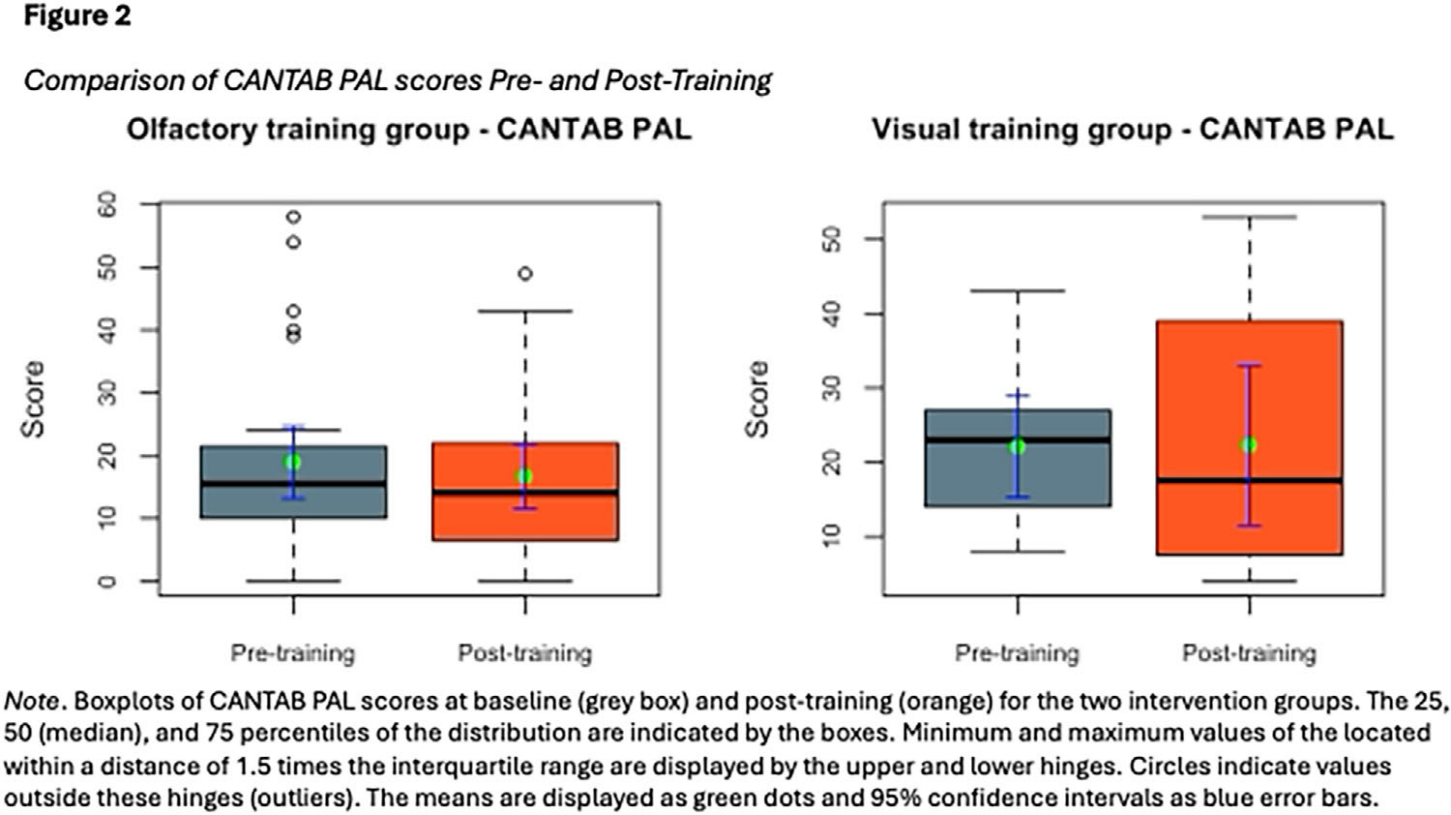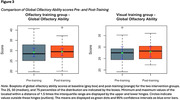# Mind Your Nose: Olfactory memory training in older adults with subjective cognitive decline

**DOI:** 10.1002/alz.095001

**Published:** 2025-01-09

**Authors:** Isabelle J.M. Burke, Christopher P.K. Brown, Courtney Chesser, Jonas Olofsson, Kate Laver, Benjamin M. Hampstead, Alex Bahar‐Fuchs

**Affiliations:** ^1^ Deakin University, Melbourne, VIC Australia; ^2^ Stockholm University, Stockholm Sweden; ^3^ Flinders University, Adelaide, SA Australia; ^4^ University of Michigan, Ann Arbor, MI USA

## Abstract

**Background:**

Memory training is associated with improved performance on memory tasks among cognitively healthy older people and those with subjective cognitive decline (SCD; Hill et al. 2017), however, demonstration of cross‐modal generalisation has been less consistent. Preliminary evidence suggests that training olfactory memory in young adults leads to the transfer of gains to visual memory tasks (Olofsson et al 2020). The Mind Your Nose trial sought to replicate this finding in older people with SCD.

**Method:**

Participants (N = 52; 17 male; Mage = 73.02, SD = 6.37) were randomly assigned to an olfactory memory training (OMT; n = 34) or a visual memory training (VMT; n = 18) intervention daily for 20 days. Outcomes included performance on the OMT and VMT tasks post training, as well as performance on a standardised measure of global olfactory ability (Sniffin Sticks; Hummel et al. 1997), and a standardised measure of visuospatial memory (CANTAB Paired Associates Learning (PAL)).

**Result:**

The OMT group showed a small, albeit non‐significant improvement on the OMT task (*M_diff_* = 14.42 (*SEM* = 8.56), Hedges *g* = 0.29, ns) and a moderate improvement on the VMT task (*M_diff_* = 15.42 (_SEM_ = 6.22), Hedges *g* = 0.43, *p* < .05). The VMT group showed a moderately large improvement on the VMT task (*M_diff_* = 31.53 (*SEM* = 11.12), *p* < .05, Hedges *g* = 0.69), but no improvement on the OMT task (*M_diff_* = ‐5.00 (*SEM* = 11.39), Hedges *g* = ‐0.11, ns). Neither group improved in global olfactory ability (OMT group: *M_diff_* = ‐0.23 (*SEM* = 1.11), Hedges *g* = ‐0.04, ns; VMT group: *M_diff_* = ‐0.57 (*SEM* = 1.11), Hedges *g* = ‐0.12, ns). The CANTAB PAL showed no improvement in the OMT group (*M_diff_* = 2.29 (*SEM* = 2.13), Hedges *g* = 0.20, ns) or the VMT group (*M_diff_* = ‐0.17 (*SEM* = 3.61), Hedges *g* = ‐0.01, ns).

**Conclusion:**

Findings from Mind Your Nose replicate previous findings from healthy young adults, supporting evidence that training memory through the olfactory modality has a greater potential for transfer to visuo‐spatial domains when compared to visual memory training, which demonstrated no transfer effects.